# Enhanced production of erythritol by *Yarrowia lipolytica* on glycerol in repeated batch cultures

**DOI:** 10.1007/s10295-013-1380-5

**Published:** 2013-11-27

**Authors:** Aleksandra M. Mirończuk, Joanna Furgała, Magdalena Rakicka, Waldemar Rymowicz

**Affiliations:** Department of Biotechnology and Food Microbiology, Wrocław University of Environmental and Life Sciences, Chełmońskiego 37/41, 51-630 Wrocław, Poland

**Keywords:** Erythritol, *Yarrowia lipolytica*, Pure glycerol, Crude glycerol, Repeated batch culture

## Abstract

Erythritol is an important natural sweetener, industrially produced only by fermentation on glucose media. Glycerol is an important renewable feedstock as it is the major by-product of the biodiesel production process; here we present an alternative way to convert this low-cost substrate into value-added products, such as erythritol. Repeated batch cultures (RBC) were performed to improve the productivity of erythritol from pure and crude glycerol. An acetate negative mutant of *Yarrowia lipolytica* Wratislavia K1 was found to be applicable for the production of high amounts of erythritol in RBC. When 20 % of fresh replaced medium was added, the strain Wratislavia K1 was able to produce 220 g l ^−1^ erythritol, which corresponded to a 0.43 g g^−1^ yield and a productivity of 0.54 g l^−1^ h^−1^. Additionally, the activity of the culture remained stable for more than 1,000 h, i.e., 11 cycles of the repeated batch bioreactors.

## Introduction

Erythritol is a four-carbon polyhydric alcohol which occurs widely in nature as a metabolite or storage compound in seaweeds or fungi, and as a component of fruits such as pears, melons, and grapes [2, 24]. This polyol as a natural sweetener has 60–70 % of the sweetness of sucrose in 10 % (w/v) solution [8]. Owing to its mouthfeel enhancing and sweetness, it has been used in the food industry since 1990. Erythritol is noncarcinogenic, noncaloric, cannot be fermented by the bacteria causing dental caries, and therefore it might be safely used in the food and pharmaceutical industries [24, 26]. In the human body, erythritol is excreted in the urine, without changing the insulin level; thus, it is also safe for diabetics [10]. Growing demand for this product is observed every year; hence erythritol production by biotechnological methods is becoming increasingly important. Fermentation processes are used for this purpose [22].

Currently, for economic reasons, research is focused on the biosynthesis of erythritol from alternative sources of carbon. The main problems involved in the biotechnological processes are reduction of production costs of erythritol and enhancing the efficient product biosynthesis. These difficulties might be solved by screening of the microorganism strains able to produce erythritol in minimal medium with a high productivity [11, 19]. Additionally, to increase the biotechnological potential of microorganisms, many researchers perform a mutagenization of selected strains [12, 33, 41]. The other method is a supplementation of media with different compounds such as manganese or copper ions, which have been found to be crucial in erythritol biosynthesis [15, 17]. For these reasons, there is increasing interest in the production of polyols from crude glycerol by oleaginous yeast *Yarrowia lipolytica*. Crude glycerol is the main by-product of biodiesel production; however, it contains many contaminants that significantly decrease its value. The purification process of crude glycerol is time and energy consuming, as it requires many procedures such as refining through filtration or fractional vacuum distillation [20]. Despite the high contamination, crude glycerol might be easily utilized by *Y. lipolytica* [28, 32, 34]. Previously it was shown that *Y*. *lipolytica* is able to convert pure or crude glycerol to organic acids, polyols, or single cell oil (SCO) in different cultivation systems [6, 18, 23, 27, 35, 37].

The most common cultivation systems used in the production of erythritol are various modifications of the batch or fed-batch cultivations [4, 19, 25, 29, 38]. The fed-batch cultures are very promising systems, easy to use for pilot experiments, verifying media compounds or supplements, and for establishing optimal technological conditions of the process. However, compared with the traditional batch cultivations, the repeated batch culture (RBC) allows for better dynamics and higher efficiency of the biosynthesis process by extending the effective production phase [37]. Up to now, the literature lacks reports on the use of RBC for the biosynthesis of erythritol by *Y*. *lipolytica* in media containing glycerol as the sole carbon source. The main aims of this research were to determine the kinetics of the technological parameters of the test process and to evaluate the effectiveness and efficiency of erythritol biosynthesis from glycerol by *Y*. *lipolytica* Wratislavia K1 in RBC.

## Materials and methods

### Microorganism

The strain used in this study was *Y*. *lipolytica* Wratislavia K1 (acetate negative mutant) with a smooth colony phenotype, which belongs to the Department of Biotechnology and Food Microbiology at Wroclaw University of Environmental and Life Sciences, Poland.

### Substrates

Pure glycerol (purity 98 % (wt wt^−1^); POCH, Poland) and crude glycerol from a biodiesel production unit (glycerol content 76 % (wt wt^−1^); LOTOS, Poland) were used as substrates. The impurities in the industrial glycerol solution were sodium salts 7 % (wt wt^−1^), 2.68 % matter organic non-glycerol (MONG), methanol 0.01 % (wt wt^−1^), metals (Cu 0.2, Mg 90, Fe 14.7, Zn 3.5 and Ca 40 ppm), heavy metals (Cd, Cr, Hg not detected), and water 13 % (wt wt^−1^).

### Media

The growth medium for inoculum preparation contained 50 g glycerol l^−1^, 3 g yeast extract l^−1^, 3 g malt extract l^−1^, and 5 g Bacto peptone l^−1^. For the first 196 h, erythritol production was conducted in a medium consisting of 100 g glycerol l^−1^, 2.3 g (NH_4_)_2_SO_4_ l^−1^, 1 g MgSO_4_·7H_2_O l^−1^, 0.23 g KH_2_PO_4_ l^−1^, NaCl 26.4 g l^−1^, and 1 g yeast extract l^−1^. After 24 h of cultivation, pure glycerol or crude glycerol solution (76 % wt wt^−1^) was added (at a constant feeding rate of 3 g h^−1^) until a total concentration of 250 g glycerol l^−1^ was obtained. After utilization of the glycerol by yeasts in a fed-batch culture, a portion of the culture (0.8, 0.6, or 0.4 l) was withdrawn, and the same volume of the production medium was added. This procedure was repeated four or three times for each volume. The replaced medium contained 250, 333.3, or 500 g glycerol l^−1^ (when 0.8, 0.6, or 0.4 l of the replaced medium was added), 2.3 g (NH_4_)_2_SO_4_ l^−1^, 1 g MgSO_4_·7H_2_O l^−1^, 0.23 g KH_2_PO_4_ l^−1^, and 1 g yeast extract l^−1^. The volume of culture broth at the start of each cycle of RBC was 2 l, and the concentration of glycerol was 100 g l^−1^. In RBC, the end of each cycle was determined when the concentration of glycerol was below 0.5 g l^−1^ and an appropriate volume of replaced medium was added.

### Culture conditions

An inoculation culture was grown in a 300-ml flask (containing 100 ml of growth medium) on a shaker at 30 °C for 3 days. An inoculum of 200 ml was introduced into the fermenter containing 1.8 l of the production medium. Fed-batch and all RBCs were performed in a 5-l jar fermenter (Biostat B Plus, Sartorius, Germany) with a working volume of 2 l at 30 °C. The aeration rate was fixed at 0.6 l min^−1^. The stirrer speed was adjusted to 800 rpm, and the dissolved oxygen concentration was maintained at 25 ± 5 % saturation. The pH was maintained automatically to 3.0 by the addition of NaOH (20 % w v^−1^). To account for dilution due to addition of NaOH solution to maintain a stable pH of the medium, amounts of polyols, biomass, and citric acid in the culture liquid were used for calculations.

### Analytical methods

Samples (10 ml) from the RBCs were centrifuged (10 min; 4 °C; 2,800×*g*), harvested by filtration on 0.45-μm-pore-size membranes, and washed twice with distilled water. The biomass was determined gravimetrically after drying in a drier at 105 °C. Concentration of glycerol, erythritol, arabitol, mannitol, α-ketoglutaric and citric acids were determined by HPLC using a HyperRez carbohydrate H+ column (Thermo Scientific, Waltham, MA) coupled to a UV (*k* = 210 nm) (Dionex, Sunnyvale, USA) and a refractive index (RI) detector (Shodex, Ogimachi, Japan). The column was eluted with 25 mM of trifluoroacetic acid (TFA) at 65 °C and a flow rate of 0.6 ml min^−1^. Protein concentration in the biomass harvested at the end of the cultivation processes was analyzed using the Kjeldahl method. The total intracellular lipids were determined according to the Soxhlet method. Fat was extracted using a Büchi B-811 universal extraction system (Büchi Labortechnic AG, Flawil, Switzerland). The fatty acid profile was determined in fat extracted from yeast biomass by modified method described by Ackman [1]. A 0.2-g sample of dry biomass was treated with a mixture of 1 ml benzene and 1 ml BF_3_–methanol. The sample was shaken for 5 min, heated for 15 min at 50 °C, and then cooled. After addition of 0.5 ml of H_2_O the sample was centrifuged and the upper layer was evaporated in a nitrogen atmosphere. The dry residue was dissolved in 0.1 ml CH_2_Cl_2_. Fatty acid methyl esters were separated by gas chromatography according to the methodology described elsewhere [16].

### Calculation of fermentation parameters

To take into account the medium dilution due to the addition of NaOH solution for maintaining the constant pH value, the amounts of erythritol and by-products in the culture broth were used for calculations of the mass yield of erythritol (*Y*_ERY_), and the volumetric erythritol productivity (*Q*_ERY_) in the RBC.

Mass yield of erythritol (*Y*_ERY_), expressed in grams per gram from glycerol, was calculated from

$$Y_{\text{ERY}} = P/S.$$

The volumetric erythritol productivity (*Q*_ERY_), expressed in grams per liter per hour, was calculated from

$$Q_{\text{ERY}} = P/ \, V \times \, t,$$

where *P* is the amount of erythritol in the culture liquid at the end of a cultivation (in grams), *S* is the total amount of glycerol consumed (in grams), *V* is the initial volume of culture liquid (in liters), and *t* is the fermentation duration (in hours).

## Results and discussion

### Strain selection

In this report, we determined the ability of *Y*. *lipolytica* Wratislavia K1 to produce erythritol from glycerol in an RBC. According to Makri et al. [21] glycerol is an appropriate substrate for the biosynthesis of many variable metabolites by *Y*. *lipolytica*. In our previous work we found that strain *Y*. *lipolytica* Wratislavia K1 was able to produce a high amount of erythritol from crude glycerol in batch and fed-batch cultures [32]. The process was conducted under osmotic stress and at low pH (3.0), which is of great importance for the industrial applications because it avoids bacterial contamination [3]. For this reason, we chose Wratislavia K1 strain for further experiments as a potential candidate for the industrial producer of erythritol.

In light of the aforementioned discussion, first we employed *Y*. *lipolytica* Wratislavia K1 strain in the fed-batch cultivation to form the first stage of erythritol production from pure and crude glycerol. The processes were conducted until the substrate was in the medium (data not shown). Subsequently, the main object of the analysis, i.e., RBC, was performed. In the RBC, we investigated the effect of fresh medium addition during the process on the erythritol production. The medium portions, which were 40, 30, and 20 % (v/v) of the culture, were replaced by the same volume of fresh medium. Each replacement was repeated three or four times; subsequently the cultures were grown under the same conditions. Consequently, we studied the influence of the medium exchange volume on the end yield and the volumetric productivity of erythritol; moreover biosynthesis of other by-products such as mannitol, citric acid, and α-ketoglutaric acid was tested (Table 1). Finally, we analyzed the influence of the medium exchange volume on the biomass production. After each cycle of exchange the biomass of *Y*. *lipolytica* Wratislavia K1 was collected to verify the accumulation of intracellular protein and fat, including the fatty acids composition (Table 2).

**Table 1:** Effect of the amount of replaced medium on biomass, erythritol, by-products, intracellular protein, and fat concentrations in RBCs of *Y. lipolytica* Wratislavia K1 strain grown in a medium containing glycerol at pH 3.0

Substrate	Replaced volume (%)	Biomass (g l^−1^)	Erythritol (g l^−1^)	Mannitol (g l^−1^)	Arabitol (g l^−1^)	Citric acid (g l^−1^)	α-Ketoglutaric acid (g l^−1^)	*Q*_ERY_ (g l^−1^h^−1^)	*Y*_ERY_ (g g^−1^)	Protein (%)	Fat (%)
Pure glycerol	40	20.3 ± 2.8	135.5 ± 6.9	3.9 ± 2.4	0.1 ± 0.2	1.1 ± 0.3	ND	0.72 ± 0.08	0.56 ± 0.02	21.3	26.8
	30	19.3 ± 2.8	174.8 ± 11.6	0.6 ± 0.6	0.1 ± 0.2	1.2 ± 0.6	0.3 ± 0.2	0.73 ± 0.08	0.54 ± 0.05	21.3	25.8
	20	20.2 ± 1.6	208.0 ± 12.0	0.2 ± 0.05	ND	0.52 ± 0.1	ND	0.44 ± 0.10	0.41 ± 0.02	21.2	23.6
Crude glycerol	40	19.5 ± 2.0	133.6 ± 6.7	3.8 ± 2.2	0.1 ± 0.1	2.2 ± 1.4	1.4 ± 1.1	0.59 ± 0.08	0.55 ± 0.07	21.0	24.8
	30	23.9 ± 2.9	110.5 ± 29.2	ND	ND	0.1 ± 0.1	ND	0.25 ± 0.2	0.27 ± 0.09	16.9	26.4
	20	21.7 ± 4.1	155.5 ± 25.1	8.1 ± 1.1	0.64 ± 0.6	5.4 ± 0.3	4.5 ± 1.7	0.30 ± 0.06	0.56 ± 0.10	23.4	26.1

*Q*_ERY_ erythritol volumetric productivity, *Y*_ERY_ erythritol yield, *ND* not detected

**Table 2:** Fatty acid composition [percent of total cellular lipids (TCL)] of *Y. lipolytica* Wratislavia K1

Exchange volume	Pure glycerol (%)			Crude glycerol (%)		
	20	30	40	20	30	40
C10:0	Traces	Traces	ND	Traces	Traces	Traces
C12:0	Traces	Traces	0.11	Traces	Traces	Traces
C14:0	0.216	0.260	0.211	0.172	0.256	0.230
C15:0	0.168	0.129	0.140	0.208	0.233	0.191
C16:0	13.775	13.076	13.599	13.236	17.485	13.980
C17:0	Traces	0.107	0.137	0.105	Traces	0.114
C18:0	4.359	3.562	4.493	4.218	5.397	3.997
C20:0	0.104	0.108	0.194	0.153	Traces	0.115
C22:0	0.229	Traces	Traces	0.129	Traces	Traces
C24:0	Traces	Traces	Traces	Traces	Traces	Traces
C16:1n7	14.086	20.606	16.488	9.174	12.485	11.373
C18:1n7	2.013	1.840	1.668	1.416	1.621	1.903
C16:1n9	4.266	4.003	4.027	3.469	2.012	3.294
C18:1n9	41.734	43.736	42.135	43.939	49.058	43.838
C20:1n9	Traces	Traces	Traces	0.121	0.124	0.169
C18:2n6	14.713	12.232	16.493	22.693	19.106	10.550
C20:2n6	Traces	Traces	Traces	Traces	Traces	Traces
C18:3n3	0.237	0.143	0.132	0.809	0.563	1.483
Total saturated fatty acids	19.046	17.322	18.926	18.352	23.562	18.790
*T*_MUFA_	62.178	70.273	64.404	58.119	65.300	60.577
*T*_PUFA_	18.776	12.405	16.670	23.529	11.138	20.633
Total unsaturated fatty acids	80.954	82.678	81.074	81.648	76.438	81.21

*T*_*MUFA*_ total of monounsaturated fatty acids,* T*_*PUFA*_ total of polyunsaturated fatty acids, *Traces* acid percentage below 0.1 %, *ND* not detected

### Production cultures

**Fig. 1:**
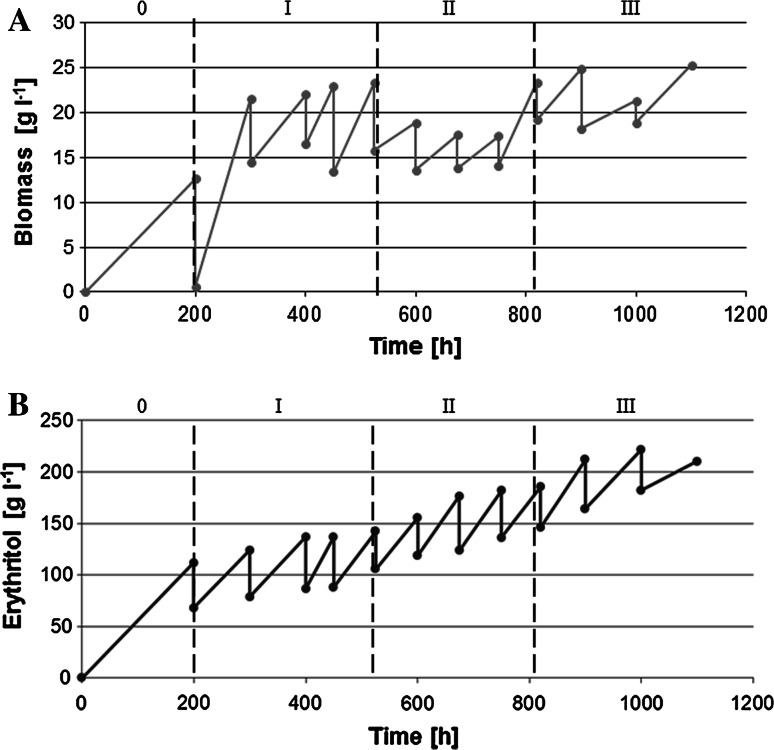
Effect of the amount of replaced medium on the biomass (**a**) and erythritol (**b**) production from pure glycerol by *Y. lipolytica* Wratislavia K1 strain during RBC mode.* 0* batch culture,* I* 50 %,* II* 40 %,* III* 30 %,* IV* 20 % medium was replaced. Each variant of RBC was repeated four or three times

**Fig. 2:**
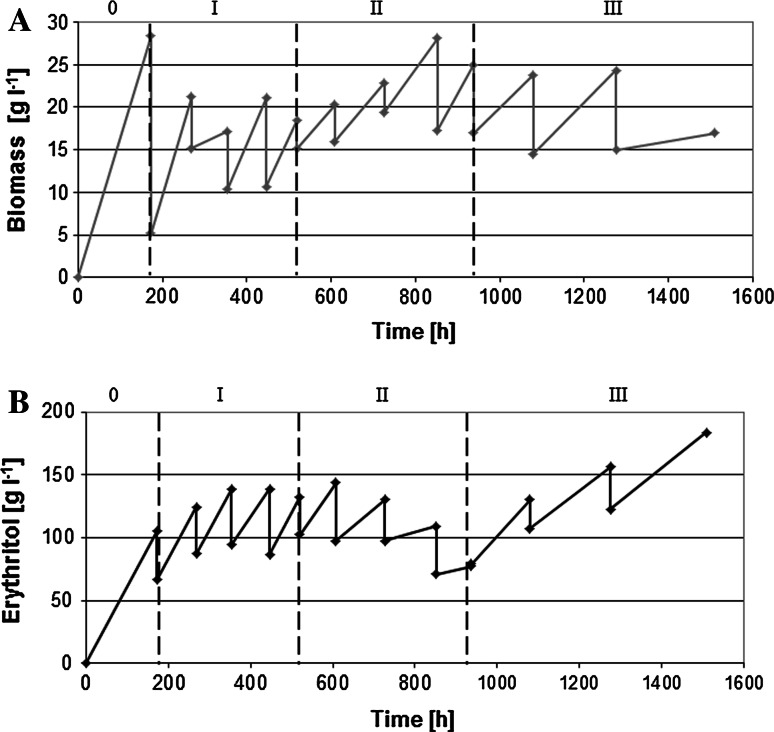
Effect of the amount of replaced medium on the biomass (**a**) and erythritol (**b**) production from crude glycerol by *Y. lipolytica* Wratislavia K1 strain during RBC mode.* 0* batch culture,* I* 50 %,* II* 40 %,* III* 30 %,* IV* 20 % medium was replaced. Each variant of RB cultures was repeated four or three times

In order to achieve high erythritol production by *Y*. *lipolytica* in the RBC, first we chose pure glycerol as the substrate, because it is free from unknown compounds, which might influence the biosynthesis process. When the conditions of the production were established, the same experiments were then performed on crude glycerol media. In our study, the RBC was proceed for 1,103 h (46 days) and 1,530 h (64 days) for pure and crude glycerol, respectively (Figs. 1, 2). During the experiments, the samples were taken every 24 h for analysis of supernatant and the growth of biomass. We noticed that multiply repeated batch experiments with two types of substrate were performed with no technical or microbiological difficulties, suggesting the high stability and feasibility of the new biosynthesis processes for erythritol production.

The main aim of our study was to investigate if RBC is an appropriate system for erythritol production from glycerol. As mentioned above, the medium exchange rates varied from 40 to 20 % (v v ^−1^). During the whole process, with addition of either pure or crude glycerol, we observed an efficient erythritol production (Table 1; Figs. 1b, 2b). Interestingly, we found that the level of erythritol biosynthesis depends on both the exchange volume rate and the type of substrate used in the RBC. Remarkably, a decrease in the amount of replaced medium resulted in higher erythritol biosynthesis. A similar effect on the desired product biosynthesis was observed in RBC during the production of citric acid by *Y*. *lipolytica* [36]. The highest production of erythritol 220 g l^−1^ with 0.41 g g^−1^ yield was obtained in the experiment with pure glycerol, when the 20 % of medium was added (Fig. 2b). Previously it was shown that *Torula* sp. is able to afford a similar product yield (0.48 g g^−1^) from glucose, but with a lower production of erythritol (192 g l^−1^) [25]. Additionally, other reports showed that the RBC system was appropriate for erythritol production; however, up to now glycerol has never been used as a substrate. Park et al. [29] described an erythritol biosynthesis by *Trichosporon* sp. on glucose syrup and corn steep liquor, where the exchange volume oscillated from 8 to 23 %. The erythritol concentration achieved 149 g l^−1^ with a yield of 0.45 g g^−1^ or 209 g l^−1^ with a yield of 0.45 g g^−1^, respectively. Furthermore, Segueilha [39] described the conversion of glucose and corn steep liquor to erythritol by yeast *Moniliella tomentosa* in an RBC that achieved a concentration of 141 g l^−1^ with a yield of 0.42 g g^−1^. In both reports, the processes were stable only for 100 h [29] or 150 h [39]. In contrast to these studies we observed that the erythritol production was stable for more than 1,000 h. Another advantage of the presented system is the use of an inexpensive medium based on glycerol, which definitely decreases the production costs. These data suggest that biosynthesis of erythritol from glycerol by *Y*. *lipolytica* is stable; therefore this system might be easier to employ in industry.

We also investigated the effect of the exchange medium on the biomass biosynthesis. It has been observed that multiple exchanges of medium with pure glycerol had no significant effect on the biomass production, which oscillated around 16.5–23.1 g l^−1^; moreover the yield of biomass remained stable during most of the process (Table 1). However, during the process with crude glycerol, the production of biomass was variable, particularly when 30 % volume of the medium was exchanged. During this step, we observed an increased yield of biomass (Fig. 2a; Table 1). Probably *Y. lipolytica* was able to utilize additional compounds such as nitrogen or phosphate, because in crude glycerol small amounts (2.68 %) of MONG were detected. Interestingly, contamination of the substrate did not have any negative influence on the growth of *Y*. *lipolytica*. Again, this unique feature of *Y*. *lipolytica* emphasizes its extraordinariness and proves the simplicity of its use for the biosynthesis of the desired compounds.

### Erythritol and by-products kinetics and yield parameters

As mentioned before, the highest concentration of erythritol production was observed when 20 % of medium was fed (Fig. 1b). For the whole process the average concentration of erythritol ranged from 128 to 220 g l^−1^. In agreement with the described results, in the RBC with crude glycerol, the average erythritol concentration oscillated from 81.3 to 180.8 g l^−1^ (Fig. 2b). Moreover the highest concentration of product was also obtained by using 20 % of the replaced medium. Although better results were obtained in the process with pure glycerol, the yield of erythritol obtained from crude glycerol is also very satisfactory. It is one of the highest levels of erythritol production described in the literature to date. Previously described studies reported the biosynthesis of erythritol mainly from glucose, which ranged from 133 to 245 g l^−1^ [9, 11–13]. The key parameters in the RBC, such as the average yield (*Y*_ERY_) and erythritol volumetric productivity (*Q*_ERY_), depend on the used substrate and the exchange volume rate. In RBC, with pure glycerol the yield of erythritol reached up to 0.56 g g^−1^; in the experiments carried out on crude glycerol the yield of erythritol reached 0.66 g g^−1^. Strikingly, the erythritol production rate described in this report is one of the highest reported in the literature. Similar yields of erythritol (0.61, 0.63 g g^−1^) were reported by Jeya et al. [13] and Lin et al. [19], respectively. Volumetric productivity of erythritol (*Q*_ERY_) obtained from pure glycerol ranged from 0.44 ± 0.10 to 0.73 ± 0.08 g l^−1^ h^−1^, whereas that for crude glycerol ranged from 0.25 ± 0.20 to 0.59 ± 0.08 g l^−1^ h^−1^ (Table 1). *Q*_ERY_ was the highest in the cycle of 40 % exchange, and it reached 0.80 or 0.67 g l^−1^ h^−1^ for pure or crude glycerol, respectively. Similar results for the biosynthesis of erythritol from glycerol were described elsewhere [40], where the productivity of erythritol oscillated above 0.86 g l^−1^ h^−1^. However, in that study, the yield of erythritol from pure glycerol was much lower and reached 0.5 g g^−1^; moreover the yield from crude glycerol was even below 0.5 g g^−1^. On the other hand, Park et al. [29] conducted the conversion of glucose to erythritol using the yeast *Trichosporon* sp.; in that report a significantly higher rate of volumetric productivity was obtained, depending on the culture system used, from 1.23 to 1.50 g l^−1^ h^−1^. Moreover, in batch cultures with *Pseudozyma* *tsukubaensis* KN75 [13] and *Moniliella* sp. N61188-12 [19] biosynthesis of erythritol from glucose also proceeded with a higher production rate, 2.84 and 1.98 g l^−1^ h^−1^, respectively. Interestingly, the authors of the aforementioned reports did not present the concentration of by-products in the supernatants. It is commonly known that high contamination of a desired product is a serious problem in the biotechnology industry. Strikingly, only in one of the mentioned reports do the authors describe high quantities of by-products such as glycerol (106.6 ± 6.6 g l^−1^) or ribitol (61.6 ± 1.8 g l^−1^) [19]. The amount of by-product was more than 50 % of all synthesized metabolites, thereby decreasing the value of the conducted processes. The possibility to obtain a pure product is crucial for the industry, because high concentrations of by-products with similar chemical structure increase the cost of purification of the desired product. Moreover, the purification of supernatant rich in additional, undesired compounds requires much longer processes.

Therefore, in our study we also determined the influence of the exchange volume rate on the biosynthesis of by-products, such as mannitol, arabitol, citric acid, and α-ketoglutaric acid. We obtained the main product with very low amounts of the by-products. During both processes, either with pure or crude glycerol, the level of by-products was less than 10 % of all the synthesized metabolites. The lowest concentration (0–0.53 g l^−1^) of by-products was observed by replacement of 20 % medium with pure and 30 % medium with crude glycerol. Arabitol and α-ketoglutaric acid were not detected in the samples during these exchange rates. The highest concentration of by-products was observed during the exchange of 20 % medium with crude glycerol: mainly mannitol 8.10 ± 1.10 g l^−1^, citric acid 5.40 ± 0.3 g l^−1^, and α-ketoglutaric acid 4.5 ± 1.7 g l^−1^. For the process carried out on pure glycerol the highest biosynthesis of by-products was observed at 40 % of replaced medium. Namely, the level of mannitol and citric acid oscillated in range 3.9 ± 2.4 and 1.1 ± 0.3 g l^−1^, respectively. However, the concentration of α-ketoglutaric acid and arabitol during this step was below 0.1 g l^−1^. This result clearly proved that the biosynthesis of erythritol from glycerol in RBC by yeast *Y*. *lipolytica* Wratislavia K1 is characterized by a high purity and productivity.

### Nutritional characteristic of *Y*. *lipolytica* Wratislavia K1 biomass

We then analyzed the biomass yield, protein, and fat content. Biomass yield was in fact stable during the long-term process, and differed slightly depending on the used substrate and the medium volume which was replaced. In the experiment performed on pure glycerol, the biomass concentration achieved on average 19.8 g l^−1^, whereas that on crude glycerol was 22.15 g l^−1^ (Table 1).

In this study the biomass production was lower than that observed by Rymowicz et al., who reported a higher concentration of biomass (26.5 g l^−1^) of *Y*. *lipolytica* Wratislavia K1 [31]. However, in the medium used for erythritol production, the level of nitrogen was limited; therefore we observed lower biomass growth. At the end of each replacement, the biomass was collected to assess its nutritional characteristics. The content of fat and protein in the biomass of *Y*. *lipolytica* Wratislavia K1 after the biosynthesis of erythritol was 23.6–26.8 % and 16.9–23.4 % for protein, respectively (Table 1). We also noticed a low content of the protein in the biomass. Additionally, it is noteworthy that nitrogen limitation results in high intracellular lipids formation.

Next, we analyzed the concentration of saturated (SFA), monounsaturated (MUFA), and polyunsaturated (PUFA) fatty acids in the total pool. The results are listed in Table 2. Interestingly, we found that the concentration of unsaturated fatty acids oscillated at about 80 % of the total fatty acids pool. The content of unsaturated fatty acids was stable during the whole process, with one exception. In the stage carried out on crude glycerol, when 30 % of medium was fed, we noticed a small decrease of unsaturated fatty acids content. The level of oleic acid (C18:1n9) oscillated from 41.734 to 49.058 %, depended on the exchange volume rate and substrate, and it was the major fatty acid that accumulated in the cells of *Y*. *lipolytica*. The highest level of oleic acid in the biomass was observed when 30 % of medium was replaced. A similar observation was made in others studies, such as those by Juszczyk et al., Makri et al., and Rymowicz et al. [14, 21, 30]. Palmitic acid (C16:0), palmitoleate acid (C16:1n7), and linoleic acid (C18:2n6) were also present in significant quantities (above 13 %) in the biomass. These results are in agreement with other studies with *Y*. *lipolytica* grown on glycerol [27]. In our experiments, the content of SFA did not exceed 20 % in the process with pure glycerol. In the process carried out on crude glycerol the content of SFA was also below 20 % with one exception, i.e., when 30 % of medium was fed. The differences in fatty acids composition in the biomass of *Y*. *lipolytica* have been thoroughly studied by others groups. It was found that most of the cellular lipids are synthesized at the end of exponential phase and at the beginning of the stationary growth phase, which is why it was called the lipogenic phase of growth [21]. Because during our experiments the stationary growth phase was extended by multiply medium replacement, we observed high content of lipids in the biomass.

The analysis of biomass showed that the content of SFA, MUFA, and PUFA obtained from pure and crude glycerol oscillated around the same level. In agreement with our results, high contents of unsaturated fatty acids were observed before [7, 14]; probably, an enhanced biological activity of unsaturated fatty acids causes a high glycerol uptake [27]. A significantly high quantity of PUFA, which are known to be essential in human and animal nutrition, were observed in the biomasses obtained from two substrates. Therefore biomass of *Y*. *lipolytica* Wratislavia K1 might be used as a diet supplement in animal feed. It is important to note that the European Feed Manufacturers’ Federation authorized the sale of *Y*. *lipolytica* fodder yeast produced from crude glycerol and registered them under the catalog number 00 575-EN.

In summary, we proved that RBC is an appropriate system for the efficient biosynthesis of erythritol by the yeast *Y*. *lipolytica* Wratislavia K1 from glycerol. This is a dynamic process, characterized by high purity and allowing one to obtain high levels of the product. In addition, the biosynthesis of erythritol from glycerol in the RBC using the most efficient exchange ratio resulted in a cheap and more environmentally friendly alternative to the currently used industrial biotechnological processes.

## Conclusions

Nowadays, an important problem facing developed countries is the growing obesity of their populations. At the present time people consume too much fat and carbohydrates that results in a higher body weight and consequently many diseases such as diabetes or cardiovascular disease [5]. The easiest way to avoid these problems is the limitation of monosaccharides and disaccharides in the human diet. A good candidate substitute for sucrose is erythritol, a four-carbon polyol, which cannot be digested by humans. For this reason, demand for erythritol has been growing over many years. Moreover, the growing world population requires more and more energy sources; therefore the production of biodiesel has increased. One important renewable product obtained in the biodiesel production is crude glycerol, which must be purified before it can be used in industry.

This study showed that RBC is an appropriate system for the erythritol biosynthesis from pure or crude glycerol by *Y*. *lipolytica* Wratislavia K1. The processes were stable for more than 1,000 h; moreover, for both types of substrate, we did not observe any technical problems. Additionally, the cultures were grown at a low pH, which protects against bacterial contamination. Another advantage of this system is a high purity of production. The concentration of by-products oscillated between 0 and 10 % of all synthesized metabolites, which definitely helps in the purification of the desired product.

Owing to the very promising results obtained with the use of RBC for erythritol production, further studies will focused on increasing the process parameters with use of crude glycerol. In these times in which care about the natural environment is paramount, the utilization of waste with its safe valorization should be a priority for scientists.
